# Secondary patellar resurfacing after total knee arthroplasty: Functional but not clinical score improvements in a retrospective multicenter study

**DOI:** 10.1002/jeo2.70501

**Published:** 2025-10-31

**Authors:** Hannes Vermue, Caroline Debette, Martial Metrop, Sarah Lyoussi, Cécile Batailler, Elvire Servien, Sébastien Lustig, LySKS (Lyon School of Knee Surgery)

**Affiliations:** ^1^ Department of Orthopedic Surgery and Sport Medicine, Croix‐Rousse Hospital FIFA Medical Center of Excellence Lyon France; ^2^ Department of Orthopedic Surgery Ghent University Hospital Ghent Belgium; ^3^ Université de Lyon, Université Claude Bernard Lyon 1 IFSTTAR LBMC UMR_T9406 Lyon France; ^4^ EA 7424, Interuniversity Laboratory of Human Movement Science Université Lyon 1 Lyon France

**Keywords:** anterior knee pain, knee society score, patient satisfaction, secondary patellar resurfacing, total knee arthroplasty

## Abstract

**Purpose:**

The role of secondary patellar resurfacing (SPR) in managing persistent anterior knee pain following total knee arthroplasty (TKA) without prior patellar resurfacing remains controversial. While primary patellar resurfacing is routine in some regions, others adopt a selective approach, leading to variability in patient outcomes and management strategies.

**Methods:**

This retrospective multicenter study analysed patients who underwent SPR after primary TKA between 2012 and 2022. Inclusion criteria encompassed patients with refractory anterior knee pain after an unresurfaced primary TKA, with at least 6 months of follow‐up. Pre‐ and postoperative knee society scores (KSS), range of motion, radiographic parameters and patient satisfaction were assessed. Thirty‐four patients (mean age: 69.8 ± 9.3 years, 68% women) met the inclusion criteria. Preoperatively, 94% of patients reported dissatisfaction.

**Results:**

Following SPR (*n* = 30), KSS function improved significantly (70.0 [interquartile range, IQR]: 60.0, 80.0) preoperatively to 80.0 (IQR: 63.8, 100.0; *p* = 0.03) postoperatively), while KSS Knee did not show a statistically significant change (*p* = 0.20). Overall, 81% of patients were satisfied or very satisfied postoperatively (*p* < 0.001). There was no significant association between preoperative patellofemoral osteoarthritis severity and postoperative KSS knee or KSS function. The complication rate requiring reoperation was 10%.

**Conclusion:**

SPR may provide meaningful clinical improvements in select patients with persistent anterior knee pain following TKA. A thorough diagnostic workup, including imaging and clinical assessment, is crucial for identifying suitable candidates. Future studies should focus on refining patient selection criteria and standardising indications for SPR.

**Level of Evidence:**

Level IV.

**Abbreviations**
BMI: body mass index
CT: computed tomography
IQR: interquartile range
KSS: knee society score
MRI: magnetic resonance imaging
OA: osteoarthritis
SPR: secondary patellar resurfacing
TKA: total knee arthroplasty

## INTRODUCTION

Persistent pain after total knee arthroplasty (TKA) remains a substantial challenge, with up to 20% of patients reporting dissatisfaction despite technically successful procedures [11, 20]. One of the most debated aspects of TKA is the management of the patella. While primary resurfacing is routine in the United States, a selective approach is adopted in many European countries, leaving a considerable proportion of patients with an unresurfaced patella [6, 16]. Registry analyses consistently show that secondary patellar resurfacing (SPR) represents one of the most frequent revision procedures following primary TKA [16, 21].

The decision to perform SPR is complex because anterior knee pain is a multifactorial entity rather than a uniform diagnosis. Recent diagnostic frameworks emphasise the need to distinguish subtypes of anterior knee pain, for example, pain aggravated by stair descent often reflects patellofemoral overload, whereas predominantly lateral anterior knee pain during flexion may indicate instability of the lateral column or flexion instability [11]. Other well‐recognised contributors include maltracking, malrotation, oversizing, residual osteophytes and iliotibial band–related syndromes [4]. Without a structured diagnostic approach, performing SPR in isolation risks poor outcomes due to overlooked causes such as instability or component malalignment. Several algorithms, such as the Bruderholz pathway, highlight the importance of systematic evaluation, including clinical history, radiographic assessment, advanced imaging (computed tomography [CT], SPECT/CT, magnetic resonance imaging [MRI]) and exclusion of infection before attributing pain to the patellofemoral joint [14].

Systematic reviews confirm that outcomes of SPR remain unpredictable, with satisfaction rates ranging from 30% to 70% [1, 5, 10, 12, 13, 15, 18, 21]. This variability is likely explained by heterogeneity in patient selection and lack of standardised diagnostic work‐up across studies. Current literature therefore stresses that anterior knee pain should be approached as a broad differential diagnosis requiring evidence‐based decision making [4, 11, 14].

The aim of this study was to evaluate the clinical outcomes, patient satisfaction and complication rates of SPR in patients with persistent anterior knee pain after unresurfaced primary TKA, and to explore whether preoperative patellofemoral osteoarthritis severity influenced these outcomes.

## MATERIAL AND METHODS

### Study design

A retrospective multicenter analysis was conducted, encompassing data from the Lyon School of Knee Surgery database from January 2012 up to June 2022. All patients who underwent SPR after primary TKA were included with at least 6 months of follow‐up. Ethics committee approval was obtained by the local Ethical Committee (2229975V0), which waived the need for written consent due to the retrospective nature of this study.

### Inclusion and exclusion criteria

**Figure 1:**
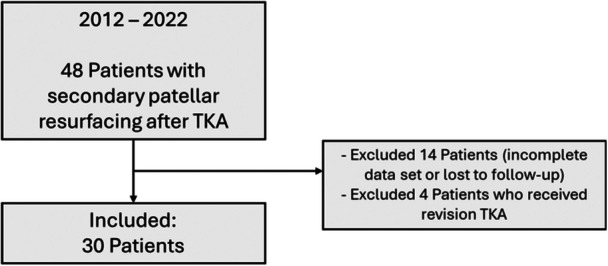
Inclusion flowchart of patients in this study. TKA, total knee arthroplasty.

Patients were included if they underwent SPR for any indication, including cases associated with other concomitant surgical procedures (such as lateral facetectomy, lateral release, polyethylene exchange, synovectomy, patellar realignment procedures). Patients who were lost to follow‐up were excluded from the study. In case patients underwent revision TKA during follow‐up, they were excluded (*n* = 4). In total 30 patients met the inclusion and exclusion criteria (Figure 1, Table 1). The current cohort consisted of mainly women (68%), with a mean age of 69.8 ± 9.3, mean body mass index (BMI) of 27.5 ± 5.7 and a mean follow‐up of 4 years. In total, 94% of patients were dissatisfied with their preoperative state, with KSS knee of 71.5 (interquartile range [IQR]: 57.0, 79.3) and KSS Function of 70.0 (IQR: 60.0, 80.0). Based on the Iwano classification, the degree of preoperative patellofemoral osteoarthritis was well spread throughout the current patient population.

**Table 1:** Preoperative demographic, clinical and radiographic characteristics of the study cohort.

Preoperative demographics	Value
Side (left/right)	53%/47%
Gender (women/men)	68%/32%
Body mass index (kg/m²)	27.5 ± 5.7
Age at secondary resurfacing of patella (years)	69.8 ± 9.3
Time to secondary resurfacing of patella (years)	2.7 ± 3.5
Follow‐up (years)	4.0 ± 2.6
Mobility (°)	
– Flexion	118.8 ± 13.2
– Extension	−0.7 ± 3.3
KSS knee score	71.5 (IQR: 57.0, 79.3)
KSS function score	70.0 (IQR: 60.0, 80.0)
Satisfaction (disappointed/dissatisfied/satisfied/very satisfied)	81%/13%/4%/2%
Patellar height (°)	0.71 ± 0.18
Patellar tilt (°)	3.7 ± 4.8
Iwano (1/2/3/4)	32.6%/23.3%/18.6%/25.6%

*Note*: Continuous variables are presented as mean ± standard deviation (SD), unless otherwise specified. Knee society scores (KSS) are presented as median with interquartile range (IQR). Patient satisfaction was self‐reported using four categories (disappointed, dissatisfied, satisfied, very satisfied). Patellar height was measured according to the Blackburne–Peel method, and patellar osteoarthritis was graded according to the Iwano classification.

### Diagnosing anterior knee pain

The technical investigations included in the work‐up for patients presenting with anterior knee pain following primary TKA is outlined in Table 2.

**Table 2:** Technical investigations performed in patients presenting with anterior knee pain after primary total knee arthroplasty (TKA).

Investigation	Indication for SPR	No indication for SPR
Clinical exam and radiograph	12 (35%)	Further investigations in 22 cases (65%)
Bone scintigraphy	19 (86%)	3 (14%)
CT‐scan	2 (33%)	4 (67%)
Aspiration	0 (0%)	2 (100%)
MRI	2 (67%)	1 (33%)

*Note*: Examinations are categorised according to whether they directly supported an indication for secondary patellar resurfacing (SPR) or not. Values are presented as absolute numbers with percentages in parentheses. In cases where the clinical examination and plain radiographs were inconclusive, additional imaging modalities were pursued. Bone scintigraphy was used to detect focal uptake in the anterior compartment; computed tomography (CT) was obtained in cases of suspected component malrotation or maltracking; aspiration was performed to exclude periprosthetic joint infection; and magnetic resonance imaging (MRI) with metal artefact reduction was applied in selected cases to evaluate cartilage status, bone marrow oedema, or occult patellar fracture.

The decision‐making process for evaluating anterior knee pain after primary TKA was structured as follows:

For patients with clear clinical findings (e.g., crepitus, painful catching or localised tenderness) and/or radiographic evidence of end‐stage patellofemoral osteoarthritis according to the Iwano classification, SPR was proposed. Instability was systematically assessed: with excessive anterior translation in flexion, or opening under varus/valgus stress in flexion raised suspicion of flexion instability resulting in patellofemoral overload was recorded [17]. Femoral oversizing was defined radiographically as a femoral component clearly exceeding the native anteroposterior dimension of the condyles on lateral radiographs, leading to anterior overstuffing [3]. When clinical examination and standard radiographs were inconclusive, additional investigations were performed. Bone scintigraphy was used to identify focal uptake in the anterior compartment; a well‐localized, intense hotspot was considered relevant and supportive of patellofemoral pathology. A CT scan was obtained in cases of suspected malrotation or maltracking, with diagnostic criteria including increased internal rotation of the femoral component (>3–5° relative to surgical transepicondylar axis) or abnormal patellar tilt/subluxation. Aspiration was performed in cases with diagnostic uncertainty to exclude periprosthetic joint infection. MRI with metal artefact reduction protocols was occasionally used to evaluate residual cartilage loss, bone marrow oedema, or occult patellar fractures when other tests remained inconclusive [19].

### Clinical and radiographic evaluation

Clinical outcomes were addressed using the original knee society score (KSS) preoperatively and at final follow‐up [7]. Range of motion was evaluated clinically and integrated in the KSS. Patient satisfaction was assessed subjectively, with patients self‐describing their state as very satisfied, satisfied, dissatisfied or disappointed. Complications which required the need for surgery were also recorded.

Preoperatively and at the last follow‐up consultation, a standardised radiographic assessment was performed included standing frontal and lateral images, frontal standing long leg films and an axial view of the patella at 30°. We recorded Patellar Height according to the Blackburn‐Peel method, Patellar Tilt and Patellar osteoarthritis according to the Iwano classification [2, 8]. All measurements were performed using Centricity Universal Viewer Zero Footprint software (version 6.0; GE Healthcare).

### Statistical analysis

Statistical analysis was performed using SPSS (Version 21, IBM). Continuous variables were presented as means with standard deviations, or median with IQR as suited. To investigate differences between the preoperative and postoperative situation, normality was evaluated with the Shapiro‐Wilk test and visually verified with boxplots. Homoscedasticity of the data was analysed with Levene tests. In case parametric prerequisites were met, paired student‐t tests were used to evaluate pre‐ to postoperative differences, whereas in case of nonparametric data Wilcoxon tests were used. Categorical variables were evaluated with a chi‐square test or Fisher′s Exact test. Statistical significance was set at 0.05.

## RESULTS

For the patients included in the current study cohort, the associated surgeries to SPR consisted of lateral facetectomy (*n* = 9), lateral release without facetectomy (*n* = 4), medial soft‐tissue advancement (*n* = 2), synovectomy (*n* = 5), insert exchange (*n* = 5) and anterior tibial tubercle osteotomy and medial patellofemoral ligament reconstruction (*n* = 2).

### Postoperative evaluation

KSS knee score transitioned from 71.5 (IQR: 57.0, 79.3) preoperatively, to 71.5 (IQR: 59.3, 90.0) postoperatively (*p* = 0.20; Table 3). Likewise, KSS Function score increased from 70.0 (IQR: 60.0, 80.0) preoperatively to a median of 80.0 (IQR: 63.8, 100.0; *p* = 0.03). A total of 81% of patients were either satisfied or very satisfied after their surgery, compared to 6% preoperatively (*p* < 0.001).

**Table 3:** The postoperative clinical and radiographical variables studied are presented.

Postoperative clinical assessment	Value
Mobility (°)	
‐ Flexion	119.9 ± 10.6
‐ Extension	−0.4 ± 2.0
KSS knee score	90.0 (74.5, 99.0)
KSS function score	90.0 (70.0, 100.0)
Satisfaction (disappointed/dissatisfied/satisfied/very satisfied)	17%/2%/46%/35%
Patellar height (°)	0.69 ± 0.16
Patellar tilt (°)	3.9 ± 4.8

Abbreviation: KSS, knee society score.

There was no statistically significant difference between patients with Iwano 1–2 and Iwano 3–4 classification regarding their postoperative KSS knee (65.0 [IQR: 54.0, 89.3]) and 89.0 (IQR: 70.8, 93.8; *p* = 0.13) and KSS function (80.0 [IQR: 60.0, 100.0]) and 75.0 (IQR: 70.0, 100.0; *p* = 0.71). There were no significant differences regarding pre‐ and postoperative range of motion, patellar height, nor patellar tilt (Tables 1 and 3).

The following complications required surgery: Haematoma (*n* = 1), patellar dislocation (*n* = 1), patellar fracture (*n* = 1), leading to a total of 3 out of 30 (10%). Four patients received revision TKA during the follow‐up period for aseptic loosening (*n* = 1), allergy (*n* = 1), residual pain (*n* = 2).

## DISCUSSION

This study contributes to the ongoing debate surrounding SPR following TKA. The findings of this study indicate that SPR can provide clinically meaningful improvements in satisfaction and function in carefully selected patients, even though the radiographic severity of patellofemoral osteoarthritis was not predictive of outcomes. This suggests that the decision to perform SPR should not rely on radiographic grading alone but rather on a comprehensive diagnostic work‐up. Our results therefore support the concept that persistent anterior knee pain after TKA is multifactorial, and SPR may only be effective when other potential pain generators are systematically excluded.

The results of SPR should be viewed in the context of pain after TKA, as the underlying mechanism leading to anterior knee pain in these patients remains poorly understood. However, several factors are believed to contribute, including improper patellar tracking, excessive body weight, implant‐to‐patella friction, or patellofemoral overstuffing [9, 20]. A thorough diagnostic process is crucial to exclude other possible sources of pain, such as prosthetic loosening, malrotation of the tibial or femoral components, infection, avascular necrosis, fractures, or referred pain. The goal of SPR is to eliminate the likely source of pain by resolving any coexisting mechanical issues in the patellofemoral joint. An important factor that requires further consideration is instability. Instability is the second most frequent cause of revision after infection and may present with anterior knee pain due to overload of the patellofemoral joint. While our diagnostic pathway incorporated CT, bone scintigraphy and MRI, these modalities have inherent limitations in detecting functional or dynamic instability.

The findings of our study show slightly higher rates of satisfied patients compared to existing literature with SPR, emphasising the need for careful patient selection and an evidence‐based approach [5, 10, 12, 13, 15, 18, 21]. Bhattee et al. reported only 39% satisfaction among their patient cohort receiving SPR, while Mockford et al. observed improvements in just 30% of cases [1, 12]. Similarly, Muoneke et al. found 44.4% satisfaction with postoperative KSS knee scores of 62.2 and KSS functions scores of 52.2 [13]. In contrast to these studies, the larger case series by Scheurer et al. and Parvizi et al. reported higher satisfaction rates of 74% and 66% [15, 18]. Of note is the fact that in the cohort of the latter study other surgeries were frequently associated to the SPR, potentially influencing the outcomes. In our series, 81% of patients reported being satisfied or very satisfied after SPR. This proportion appears higher than the 50–60% satisfaction rates described in larger registry‐based and cohort studies. However, these results must be interpreted with caution. The relatively small cohort size, referral patterns and heterogeneity of implant designs may have introduced selection bias and limit the generalisability of our findings. Rather than reflecting a true difference in effectiveness, these outcomes illustrate what may be achievable in carefully selected patients undergoing SPR after thorough diagnostic work‐up.

**Figure 2:**
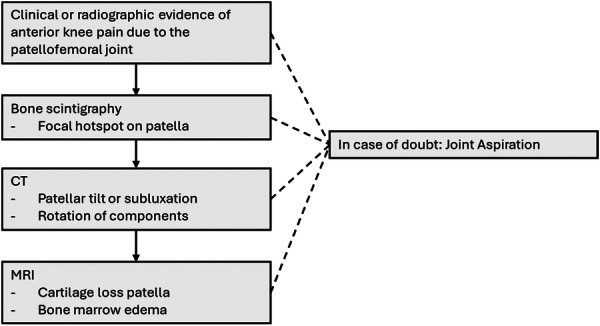
The clinical decision pathway used by the surgeons to define the indications for secondary patellar resurfacing. CT, computed tomography; MRI, magnetic resonance imaging.

That is why the lack of clear predictors for a positive surgical outcome following SPR remains a critical challenge. A registry‐based study by Thomas et al. revealed no significant association between demographic factors, such as age or gender, and successful outcomes [21]. The timing of the procedure relative to primary TKA also did not predict success, contrasting with previous studies suggesting a reduced benefit with delayed resurfacing. This is why the authors believe proper patient selection for SPR still remains essential to improve the surgical outcomes of the procedure. As a result, new studies on SPR should be encouraged to provide a systematic description of the diagnostic process leading to the indication for SPR. Based on our results, the authors propose a decision making pathway to guide surgeons diagnosing anterior knee pain, based on clinical examination, radiographic evaluation, which was complemented by more specific imaging techniques such as bone scintigraphy, CT and MRI in case the bone scintigraphy was not conclusive (Figure 2). As periprosthetic joint infections should not be overlooked, in specific cases, a joint aspiration was performed in the current case series. While performing SPR just for the sake of relieving anterior knee pain after TKA might be tempting, a systematic diagnostic work‐up like the one suggested in this study might enhance the accuracy of identifying candidates who will benefit from SPR.

Complication rates also vary with some studies, such as Garcia et al. and Mockford et al., reporting no complications at all, while others, such as Karnezis et al. and Muoneke et al., report complication rates of approximately 30% [5, 10, 12, 13]. The complication rate of our study lies in the lower end of the spectrum with a complication rate requiring revision of 8.8%.

Despite its strengths, this study has several limitations. This study shares limitations with existing literature due to its retrospective nature. The cohort size remains small, provided the rarity of SPR. Furthermore, the inclusion of associated surgeries, such as lateral release, lateral facetectomy, or polyethylene exchange, introduces potential confounders that may have influenced the surgical outcomes. These additional procedures were performed in selected cases to optimise patellofemoral mechanics or to address concomitant intra‐articular pathology, and it is therefore difficult to isolate the effect of SPR alone. While this reflects real‐world surgical practice, it also limits the ability to attribute postoperative improvements exclusively to patellar resurfacing. The cohort was heterogeneous with respect to implant designs, as multiple manufacturers and models were represented. Because many patients were referred to our centres, the original alignment strategy during the index TKA was unknown. As well, the diagnostic work‐up proposed in this study was defined based on patients who have received SPR, whereas these findings cannot be generalised to the diagnostic work‐up of a painful TKA in general. The follow‐up period was sufficient to capture stable clinical outcomes; however, its heterogeneity limits robust assessment of complication rates and implant survival. Furthermore, while the diagnostic work‐up presented in this study is descriptive rather than standardised, it reflects a pragmatic, real‐world approach across multiple surgeons and centres. Further research is warranted to clarify the role of SPR in managing anterior knee pain after TKA. Comparative studies could provide valuable insights into the relative effectiveness of SPR. Additionally, the identification of preoperative risk factors, such as quadriceps atrophy, patellofemoral dysplasia, or abnormal tracking, may help refine the selection criteria for SPR, improving surgical outcomes.

## CONCLUSIONS

Secondary resurfacing of the patella after TKA can provide meaningful improvements in pain relief and satisfaction for some patients. Although, its results are less predictable than primary patellar resurfacing, a structured diagnostic algorithm may contribute to early diagnosis and clinical outcomes higher than currently reported in literature.

## AUTHOR CONTRIBUTIONS

**Hannes Vermue, Caroline Debette**: Data curation; formal analysis; original draft. **Martial Metrop, Sarah Lyoussi**: Data curation; writing—review and editing. **Cécile Batailler, Elvire Servien, Sébastien Lustig, LySKS**: Methodology; supervision; writing—review and editing.

## CONFLICT OF INTEREST STATEMENT

Elvire Servien: Consultant for Corin. Sébastien Lustig: Consultant for Stryker, Smith and Nephew, Heraeus. Institutional research support to Lepine and Amplitude. Editorial Board for Journal of Bone and Joint Surgery (Am). The remaining authors declare no conflict of interest.

## ETHICS STATEMENT

Ethical approval was obtained by the local ethical committee (ref. 2229975V0), which waived informed consent due to the retrospective nature of the study.

## Data Availability

The data that support the findings of this study are available from the corresponding author upon reasonable request.
